# Supplementary material for: Internal Validation of MaSTR™ Probabilistic Genotyping Software for the Interpretation of 2–5 Person Mixed DNA Profiles
Source: Genes (Basel). 2022 Aug 11;13(8):1429. doi: 10.3390/genes13081429 (PMC9408203; doi:10.3390/genes13081429)
Supplement: Supplementary file 1 [file genes-13-01429-s001.zip › genes-1799783-supplementary.pdf]

Supplementary materials

Supplementary Table S1. Summary of the calculated random match probability (RMP) values for a single source donor (1692) at each locus and complete genotype using the HWE, NRC 4.1, and NRC 4.2 recommended formulae in Microsoft Excel spreadsheet and MaSTR™ to assess accuracy.

Locus	Genotype	**Excel** **HWE**	**MaSTR** **HWE**	**Excel** **(Rec. 4.1 F=0.01)**	**MaSTR** **(Rec. 4.1 F=0.01)**	**Excel** **(Rec. 4.2 θ =0.01)**	**MaSTR** **(Rec. 4.2 θ =0.01)**
D3S1358	14, 16	20.2370	2.02×10^{1}	20.2370	2.02×10^{1}	18.4094	1.84×10^{1}
D1S1656	16, 16.3	51.6087	5.16×10^{1}	51.6087	5.16×10^{1}	44.1061	4.41×10^{1}
D2S441	10, 15	62.9509	6.30×10^{1}	62.9509	6.30×10^{1}	50.0913	5.01×10^{1}
D10S1248	14, 14	11.4250	1.14×10^{1}	11.1594	1.12×10^{1}	10.1969	1.02×10^{1}
D13S317	10, 12	27.8841	2.79×10^{1}	27.8841	2.79×10^{1}	24.2145	2.42×10^{1}
Penta E	12, 16	61.5728	6.16×10^{1}	61.5728	6.16×10^{1}	50.7644	5.08×10^{1}
D16S539	9, 10	28.4365	2.84×10^{1}	28.4365	2.84×10^{1}	25.7375	2.57×10^{1}
D18S51	14, 17	29.0223	2.90×10^{1}	29.0223	2.90×10^{1}	26.3015	2.63×10^{1}
D2S1338	19, 23	28.4472	2.84×10^{1}	28.4472	2.84×10^{1}	25.7952	2.58×10^{1}
CSF1PO	10, 12	6.2505	6.25×10^{0}	6.2505	6.25×10^{0}	6.1167	6.12×10^{0}
Penta D	11, 11	41.1493	4.11×10^{1}	39.0357	3.90×10^{1}	32.0588	3.21×10^{1}
TH01	6, 6	26.0441	2.60×10^{1}	25.0175	2.50×10^{1}	21.4930	2.15×10^{1}
vWA	14, 17	19.9653	2.00×10^{1}	19.9653	2.00×10^{1}	18.2753	1.83×10^{1}
D21S11	31, 31.2	80.8163	8.08×10^{1}	80.8163	8.08×10^{1}	66.7105	6.67×10^{1}
D7S820	11, 12	15.5985	1.56×10^{1}	15.5985	1.56×10^{1}	14.6354	1.46×10^{1}
D5S818	11, 11	10.0685	1.01×10^{1}	9.8543	9.85×10^{0}	9.0732	9.07×10^{0}
TPOX	8, 8	4.6006	4.60×10^{0}	4.5485	4.55×10^{0}	4.3520	4.35×10^{0}
D8S1179	12, 13	13.1320	1.31×10^{1}	13.1320	1.31×10^{1}	12.4185	1.24×10^{1}
D12S391	19.2, 21	2056.7947	2.05×10^{3}	2056.7950	2.05×10^{3}	378.5465	3.78×10^{2}
D19S433	14, 16.2	70.9739	7.10×10^{1}	70.9739	7.10×10^{1}	50.2824	5.03×10^{1}
FGA	20, 26	114.9955	1.15×10^{2}	114.9956	1.15×10^{2}	90.0018	9.00×10^{1}
D22S1045	15, 16	5.2400	5.24×10^{0}	5.2400	5.24×10^{0}	5.1644	5.16×10^{0}
		2.73×10^{32}	2.73×10^{32}	2.35×10^{32}	2.35×10^{32}	2.90×10^{30}	2.90×10^{30}

Supplementary Table S2. Random match probabilities (RMPs) for each of the true contributor genotypes used calculated by hand and using MaSTR™ with the NRC 4.1 equation.

Contributor	Random Match Probability calculated by hand	Random Match Probability calculated by MaSTR™
1653	1.45×10^{-34}	1.45×10^{-34}
1657	8.57×10^{-35}	8.58×10^{-35}

1658	2.30×10^{-26}	2.30×10^{-26}
1659	5.39×10^{-32}	5.39×10^{-32}
1660	2.72×10^{-29}	2.72×10^{-29}
1665	7.18×10^{-37}	7.17×10^{-37}
1666	1.64×10^{-33}	1.63×10^{-33}
1668	9.57×10^{-30}	9.57×10^{-30}
1669	2.44×10^{-29}	2.44×10^{-29}
1670	9.91×10^{-31}	9.90×10^{-31}
1678	9.23×10^{-34}	9.22×10^{-34}
1679	6.06×10^{-36}	6.05×10^{-36}
1681	5.08×10^{-34}	5.08×10^{-34}
1682	3.61×10^{-31}	3.61×10^{-31}
1683	4.86×10^{-29}	4.85×10^{-29}
1686	1.52×10^{-31}	1.52×10^{-31}
1690	7.95×10^{-29}	7.95×10^{-29}
1693	4.53×10^{-30}	4.52×10^{-30}

Supplementary Table S3. Manual calculations to evaluate LR for the major contributor given the weighted genotypes from MCMC in the MaSTR™ software. Mixture 2-person_1v10m_1660_1693 (~6:63 pg) was selected to manually test concordance. The mixture had two persons with high allele sharing, and the reference (POI) was defined as genotype 1693 (alleles 10, 11; major contributor) while the minor contributor genotype 1660 (alleles 10, 14) was defined as a known. The calculations were performed without co-ancestry adjustment and incorporated the possibility of Drop-out alleles, defined as Q alleles. The results from the D2S441 locus are shown as an example analyzed using the All Data allele frequencies from the Hill *et al* database [35]. The upper panel shows the manual setup of the LR and the hand calculations for 1693 given the four combinations of genotypes with the highest weights. The lower panel shows the results from MaSTR™ for 1693 in the highlighted locus. Additional loci demonstrated concordant LRs when treated in the same way.

LR = Pr(E|Hp) / Pr(E|Hd)

Pr(E|Hp) – all the evidence can be explained by victim and person of interest genotypes

Pr(E|Hd) – evidence can be explained by victim and an unknown person (DNA originated from either 10,11 or 11,11 or 11,14 or 11,Q

Pr(E|Hd)= 0.9463 x 2 x f_{10} x f_{11} + 0.0445x f_{11} x f_{11} + 0.0092 x 2 x f_{11} x f_{14} + 0.0001 x 2 x f_{11} x f_{Q}

f_{Q} = 1 – sum of the frequencies of non-dropout alleles (alleles detected in the mixture)

1 – (0.20319 + 0.34025 + 0.22683) = 0.22973

Pr(E|Hd)= 0.130845653 + 0.005151768 + 0.001420092 + 0.0000156531

Pr(E|Hd)= 0.137433146(denominator in result table from MaSTR at top)

Pr(E|Hp) = weight of the 10,11 genotype from MCMC above (would be 1 in binary method)

LR = 0.9463 / 0.1374 =6.89

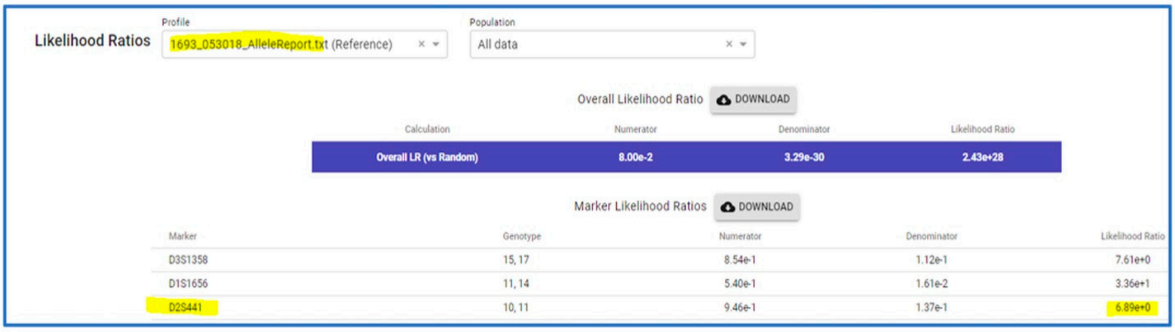

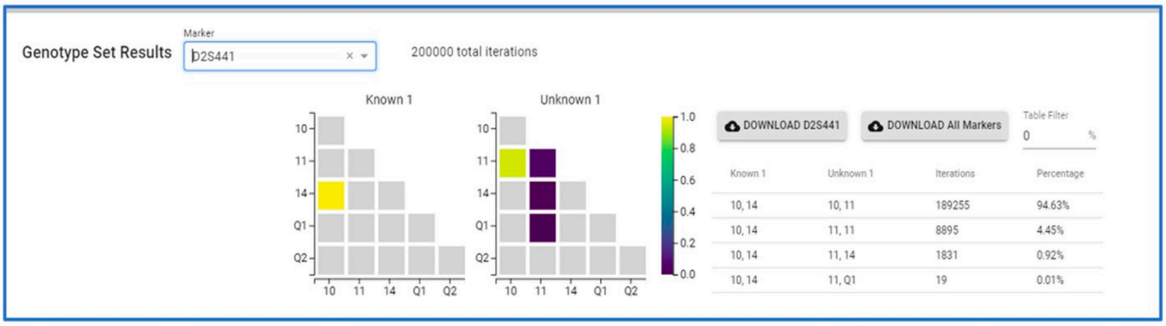

Supplementary Table S4. Comparison of LR calculations performed by hand using the MaSTR™ weighted modeled probabilities, LR calculations performed by MaSTR™, and LR calculations performed by hand using unweighted probabilities from the intuitive major contributor. Mixture 2-person_1v10m_1660_1693 (~6:63 pg) was selected to manually evaluate concordance. The mixture had two contributors with high allele sharing, and the major contributor reference (POI) was defined as genotype 1693 while the minor contributor genotype 1660 was defined as a known. The calculations with weighted probabilities were performed without co-ancestry adjustment and incorporated the possibility of Drop-out alleles, defined as Q alleles. The calculations using unweighted probabilities considered only the most intuitive major contributor.

Locus	Contributor 1693 Genotype	LR calculated by hand using	LR calculated by MaSTR™ using	LR calculated by hand using

		weighted probabilities	weighted probabilities	unweighted probabilities
D3S1358	15, 17	7.61 x 10^0	7.61 x 10^0	8.04 x 10^0
D1S1656	11, 14	3.36 x 10^1	3.36 x 10^1	6.74 x 10^1
D2S441	10, 11	6.89 x 10^0	6.89 x 10^0	7.23 x 10^0
D10S1248	14, 15	8.13 x 10^0	8.13 x 10^0	8.39 x 10^0
D13S317	11, 12	5.40 x 10^0	5.40 x 10^0	5.65 x 10^0
Penta E	12, 14	4.75 x 10^1	4.75 x 10^1	4.75 x 10^1
D16S539	9, 12	1.20 x 10^1	1.20 x 10^1	1.20 x 10^1
D18S51	14, 17	2.90 x 10^1	2.90 x 10^1	2.90 x 10^1
D2S1338	16, 20	9.40 x 10^1	9.40 x 10^1	9.40 x 10^1
CSF1PO	10, 11	7.70 x 10^0	7.68 x 10^0	7.87 x 10^0
Penta D	9, 13	1.66 x 10^1	1.66 x 10^1	1.66 x 10^1
THO1	7, 9.3	8.25 x 10^0	8.25 x 10^0	8.25 x 10^0
vWA	14, 17	2.00 x 10^1	2.00 x 10^1	2.00 x 10^1
D21S11	27, 30	5.23 x 10^1	5.23 x 10^1	5.23 x 10^1
D7S820	9, 10	1.04 x 10^1	1.04 x 10^1	1.40 x 10^1
D5S818	10, 13	3.90 x 10^1	3.90 x 10^1	3.93 x 10^1
TPOX	8, 8	4.60 x 10^0	4.60 x 10^0	4.60 x 10^0
D8S1179	13, 14	7.89 x 10^0	7.89 x 10^0	7.98 x 10^0
D12S391	23, 24	1.58 x 10^2	1.58 x 10^2	1.42 x 10^2
D19S433	12, 15.2	1.05 x 10^2	1.05 x 10^2	1.05 x 10^2
FGA	19, 24	5.88 x 10^1	5.88 x 10^1	6.30 x 10^1
D22S1045	11, 16	1.28 x 10^1	1.28 x 10^1	1.30 x 10^1
Profile LR		2.43 x 10^{28}	2.43 x 10^{28}	8.02 x 10^{28}

Supplementary Table S5. Summary of LRs calculated for the same two-, three-, and four-person mixtures using MaSTR™ and EuroForMix to assess accuracy. The listed order of each contributor indicates its position in the mixture ratio, smallest to largest. The two- and three-person mixtures show results for both low allelic sharing (1678 & 1653/1679 & 1683 & 1657) and high allelic sharing (1660 &1693/1668 & 1658 & 1659) combinations. Data shown is for undiluted mixtures and the approximate quantity of amplification DNA template in picograms is listed for each contributor. MaSTR™ values shown are overall (sub-sub-source) LRs. Analysis parameters were kept as close as possible between the two systems; only backwards (N-1) stutter was considered, no degradation options were selected, and equation NRC 4.2 was used for both.

Mixture Condition	Contributor	MaSTR™ LR	EuroForMix LR
2 Person 1:1 (~500:500 pg)	1678	5.54 x 10^{14}	5.48 x 10^{17}
	1653	2.45 x 10^{15}	1.87 x 10^{16}
2 Person 1:3 (~160:500 pg)	1678	8.13 x 10^{23}	9.11 x 10^{27}
	1653	5.26 x 10^{31}	1.58 x 10^{31}
2 Person 1:1 (~500:500 pg)	1660	9.50 x 10^{12}	2.97 x 10^{13}

	1693	7.63 x 10^{13}	1.87 x 10^{17}
2 Person 1:3 (~160:500 pg)	1660	7.46 x 10^{25}	6.30 x 10^{20}
	1693	8.87 x 10^{27}	7.12 x 10^{28}
3 Person 1:1:1 (~500:500:500 pg)	1679	3.21 x 10^{17}	7.99 x 10^{13}
	1683	9.55 x 10^{3}	4.00 x 10^{6}
	1657	1.00 x 10^{16}	6.82 x 10^{11}
3 Person 1:1:2 (~250:250:500 pg)	1679	3.60 x 10^{14}	5.12 x 10^{12}
	1683	7.54 x 10^{10}	1.46 x 10^{5}
	1657	5.82 x 10^{28}	6.62 x 10^{16}
3 Person 1:1:1 (~500:500:500)	1668	4.48 x 10^{10}	2.53 x10^{10}
	1658	2.02 x 10^{9}	9.16 x 10^{8}
	1659	2.54 x 10^{13}	2.97 x 10^{14}
3 Person 1:1:2 (~250:250:500)	1668	2.47 x 10^{12}	1.41 x 10^{9}
	1658	5.04 x 10^{10}	3.28 x 10^{8}
	1659	7.80 x 10^{19}	2.66 x 10^{15}
3 person 1:3:5 (~100:300:500 pg)	1668	2.72 x 10^{4}	2.44 x 10^{2}
	1658	1.33 x 10^{12}	7.19 x 10^{8}
	1659	7.33 x 10^{22}	5.09 x 10^{18}
4 Person 1:2:2:5 (~100:200:2000:500 pg)	1669	3.90 x 10^{10}	3.33 x 10^{4}
	1682	3.98 x 10^{15}	9.07 x 10^{8}
	1683	1.58 x 10^{9}	4.55 x 10^{5}
	1690	8.53 x 10^{24}	1.84 x 10^{14}

Supplementary Table S6. Summary of differences in average LR values (N=5 MaSTR™ runs) between three amplification replicates in a representative range of mixtures. The LRs shown are for each of the contributors to each of the mixtures with the position (*) and approximate quantity (pg) of the contributor denoted for the replicates' values. Mixture ratios were 1:1 for the two-person, 1:3:5 for the three-person, 1:1:3:10 for the four-person, and 1:2:2:5:10 for the five-person.

Mixture Condition	Amplification 1 Average LR	Amplification 2 Average LR	Amplification 3 Average LR
Contributor 1678 2 Person ~500*:500 pg	2.09 x 10^{16}	1.37 x 10^{13}	2.61 x 10^{12}

Contributor 1653 2 Person ~500:500* pg	1.34×10^{17}	8.43×10^{13}	1.72×10^{13}
Contributor 1679 3 Person ~25*:75:125 pg	1.34×10^{2}	1.34×10^{-5}	1.41×10^{-4}
Contributor 1683 3 Person ~25:75*:125 pg	1.11×10^{10}	8.65×10^{12}	4.01×10^{13}
Contributor 1657 3 Person ~25:75:125* pg	8.24×10^{28}	2.28×10^{30}	1.07×10^{31}
Contributor 1682 4 Person ~13*:13:38:125 pg	4.42×10^{2}	1.23×10^{4}	4.89×10^{7}
Contributor 1683 4 Person ~13:13*:38:125 pg	2.84×10^{4}	6.10×10^{3}	3.31×10^{0}
Contributor 1690 4 Person ~13:13:38*:125 pg	1.61×10^{13}	4.34×10^{11}	1.46×10^{15}
Contributor 1669 4 Person ~13:13:38:125* pg	5.35×10^{23}	5.64×10^{24}	4.66×10^{24}
Contributor 1665 5 Person ~13*:25:25:63:125 pg	1.96×10^{2}	6.45×10^{-1}	1.69×10^{-1}
Contributor 1681 5 Person ~13:25*:25:63:125 pg	4.88×10^{3}	1.02×10^{2}	1.03×10^{0}
Contributor 1686 5 Person ~13:25:25*:63:125 pg	2.01×10^{0}	2.18×10^{3}	3.23×10^{3}
Contributor 1666 5 Person ~13:25:25:63*:125 pg	5.36×10^{11}	4.29×10^{13}	9.41×10^{13}
Contributor 1670 5 Person ~13:25:25:63:125* pg	3.56×10^{17}	9.85×10^{15}	2.29×10^{16}

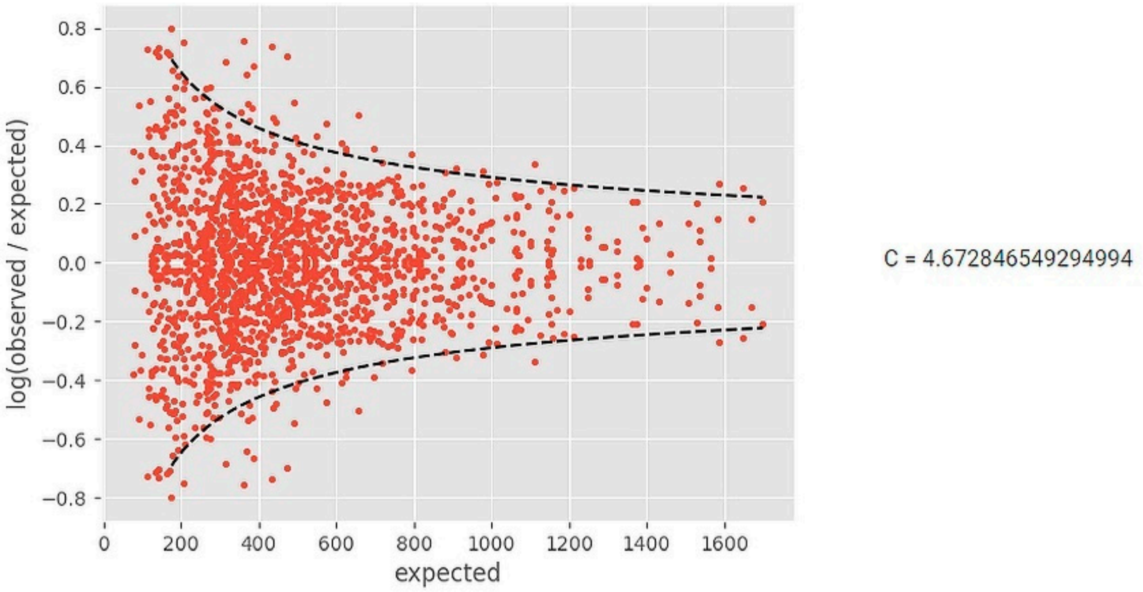

Supplementary Figure S1. MaSTR™ generated variance factor plot for 50 RFUs analytical threshold showing the expected peak heights (RFUs) on the X axis plotted against the log of the heterozygous peak balance on the Y axis.

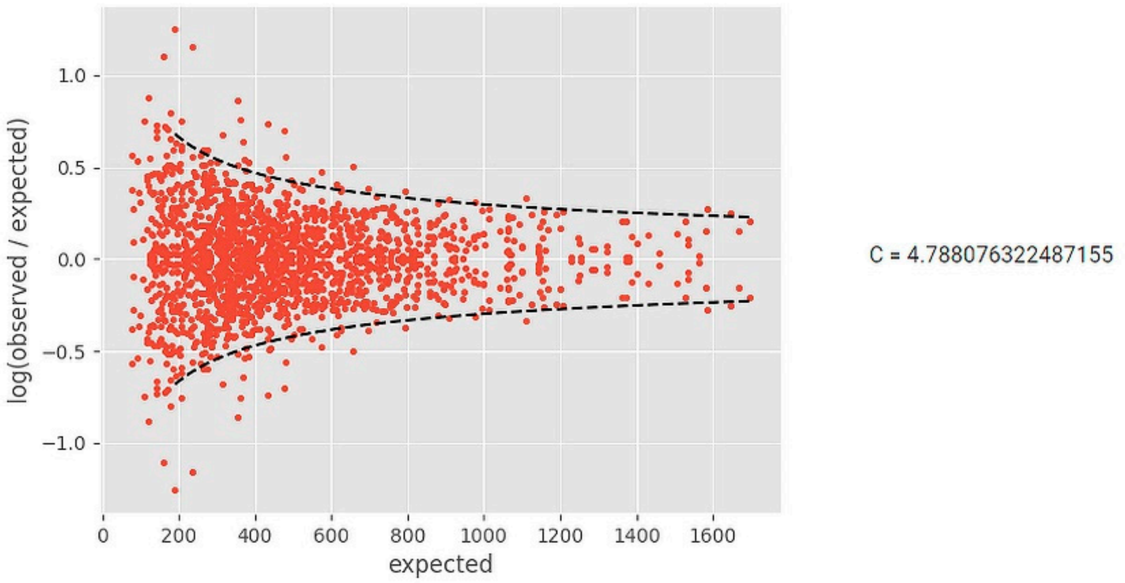

Supplementary Figure S2. MaSTR™ generated variance factor plot for 30 RFUs analytical threshold showing the expected peak heights (RFUs) on the X axis plotted against the log of the heterozygous peak balance on the Y axis.

Supplementary Figure S3a.

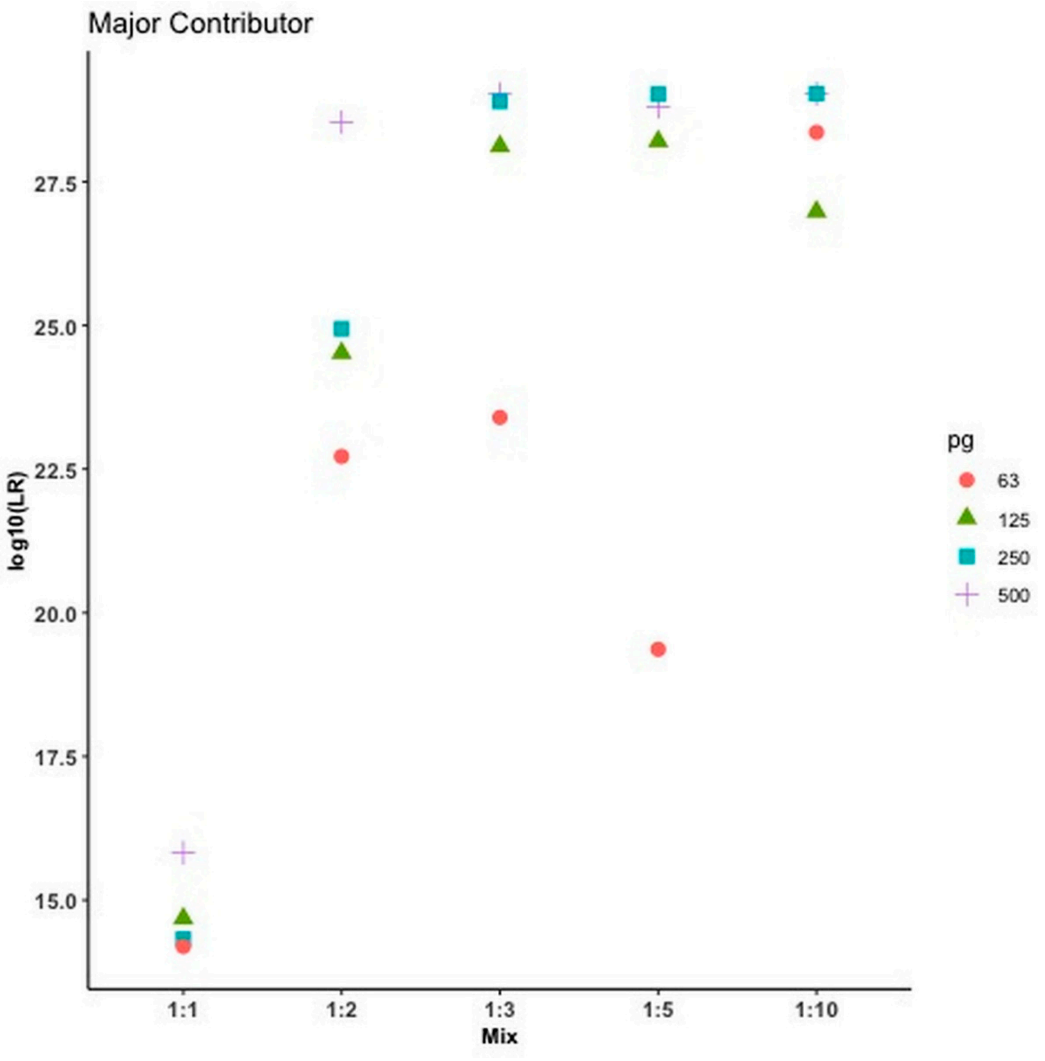

Supplementary Figure S3b.

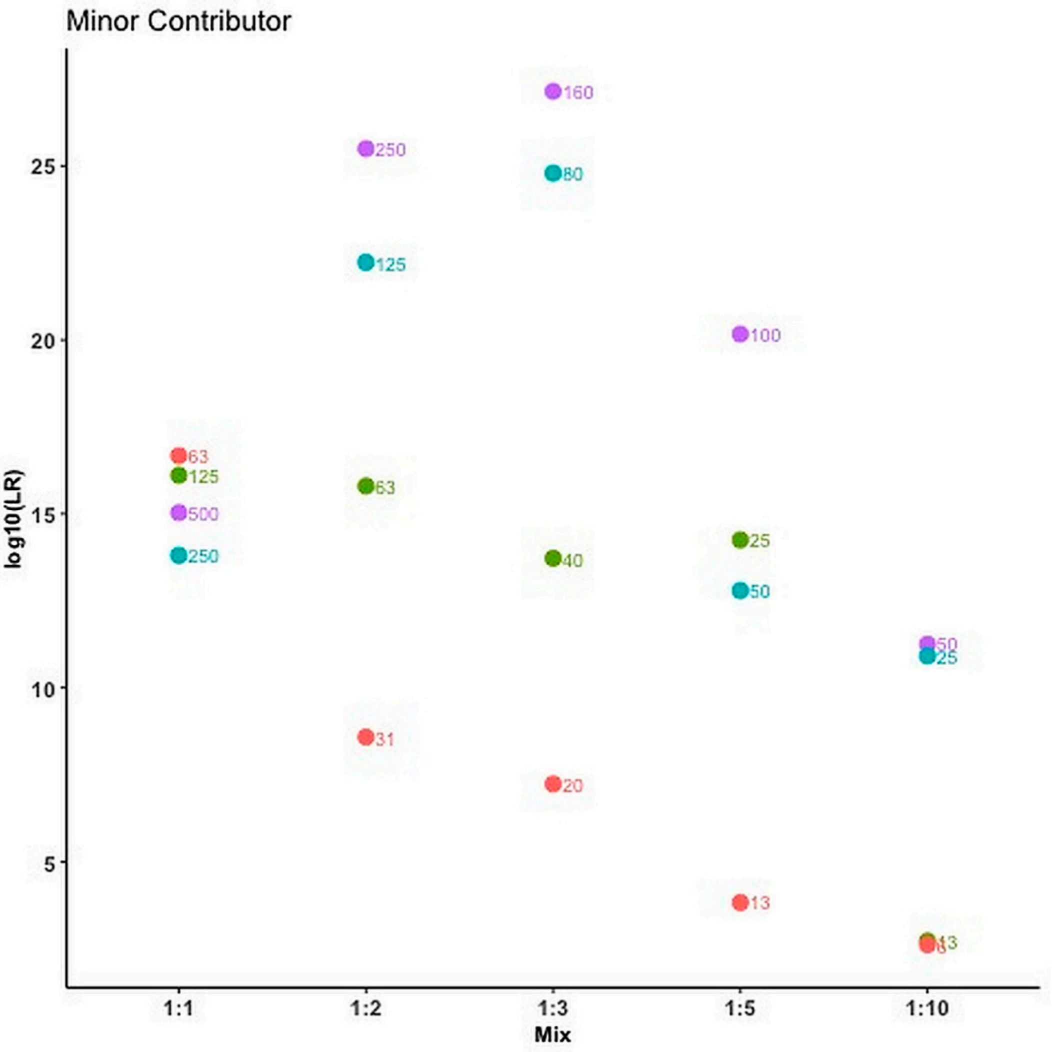

Supplementary Figure S3. Modeling performance of MaSTR™ using a two-person mixture (contributors 1660 and 1693). The average LRs (N=5) from the major contributor (1693) are shown in panel 3a and minor contributor (1660) in panel 3b. The DNA template quantities were consistent for the major contributor across the mixture ratios and are indicated in approximate picograms by color and shape on the right in panel 3a. The DNA template quantities for the minor contributor changed with the specific mixture ratio used and so are indicated in approximate picograms next to each data point

Supplemental Figure S4a.

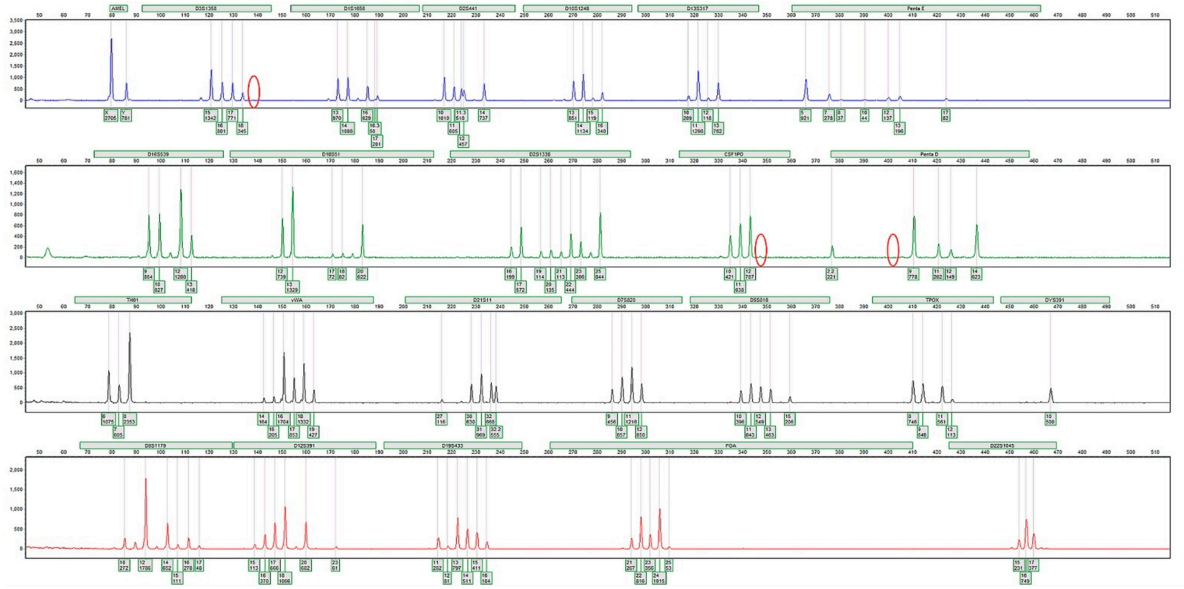

Supplemental Figure S4a.

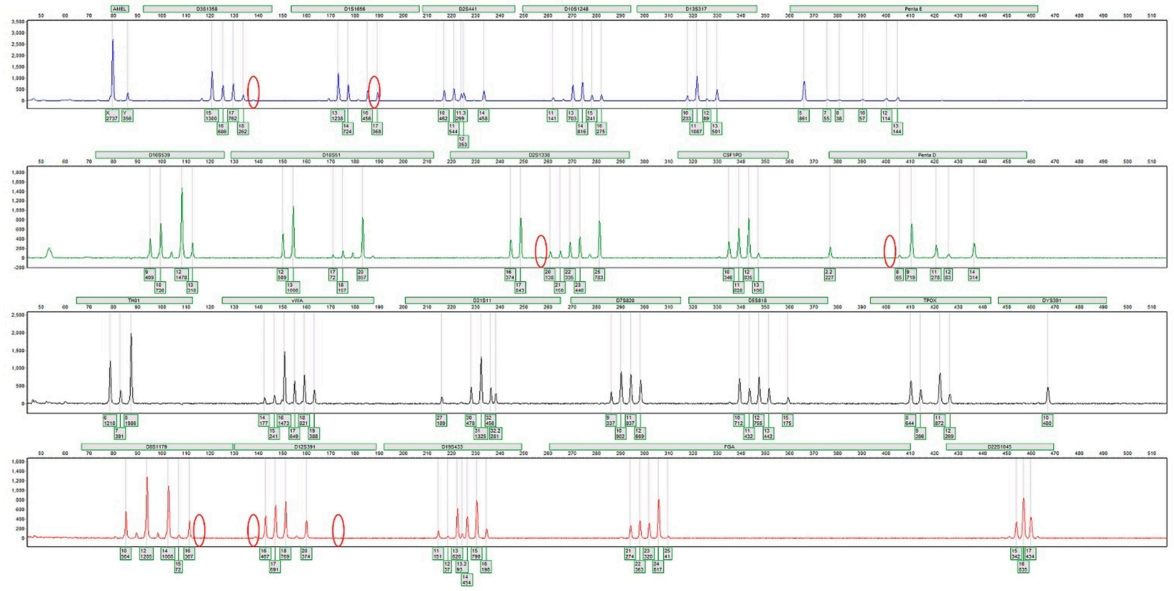

Supplemental Figure S4c.

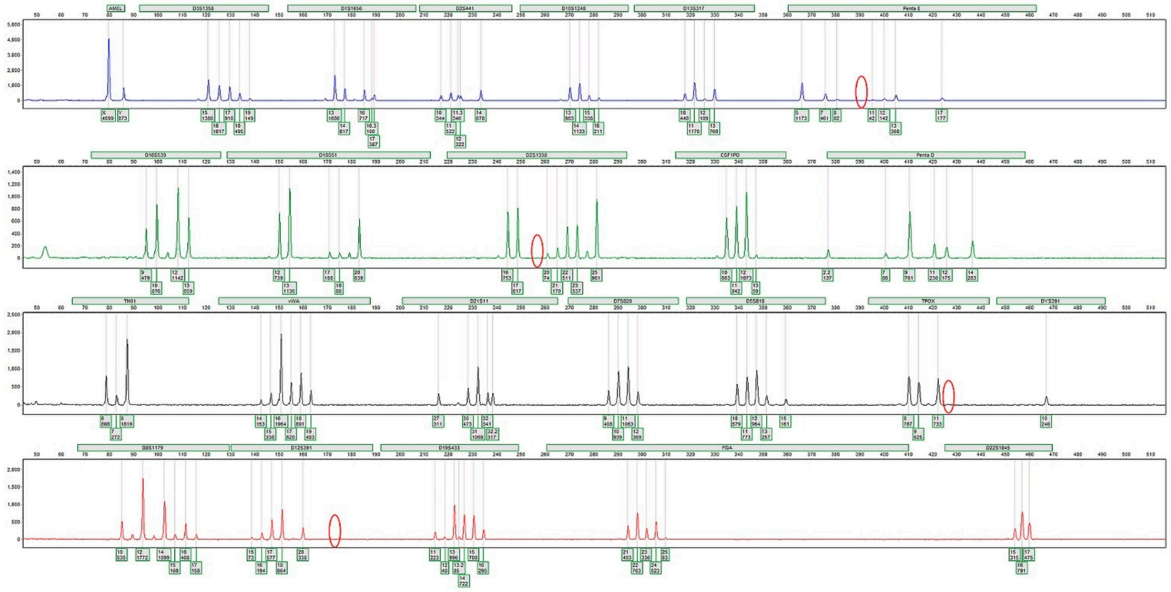

Supplementary Figure 4. Electropherograms for the five-person mixture with a contributor ratio of 1:1:5:5:10, diluted 1:2 (~25:25:125:125:250 pg). Panel 3a shows the allele peaks for amplification replicate #1. Panel 3b shows the allele peaks for amplification replicate #2. Panel 3c shows the alleles peaks for amplification replicate #3. The positions for allele drop out in contributor 1665 are indicated. Contributor 1665 full genotype: (D3S1358 16, 19), (D1S1656 14, 16.3), (D2S441 12, 12), (D10S1248 15, 15), (D13S317 11, 12), (Penta E 8, 10), (D16S539 9, 12), (D18S51 18, 20), (D2S1338 19, 21), (CSF1PO 12, 13), (Penta D 7, 9), (THO1 7, 7), (vWA 15, 15), (D21S11 27, 30), (D7S820 10, 10), (D5S818 11, 13), (TPOX 8, 12), (D8S1179 15, 17), (D12S391 15, 23), (D19S433 13, 15), (FGA 23, 25), and (D22S1045 17, 17).

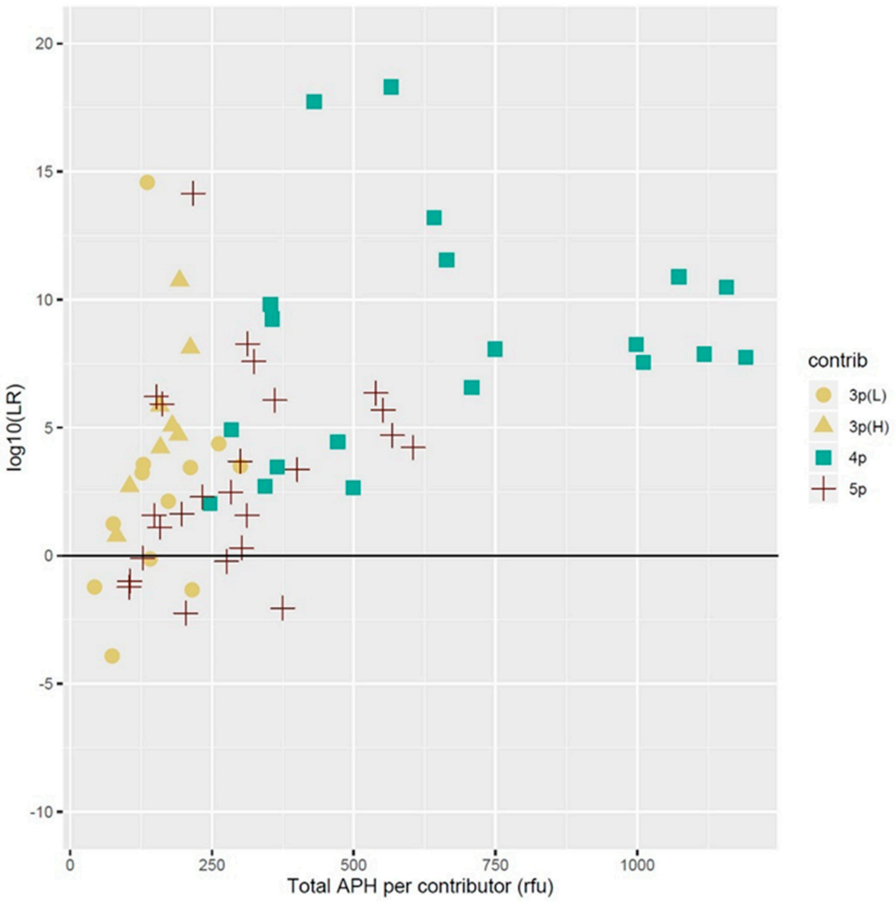

Supplementary Figure S5. The log10 of the average LRs (N=5) versus average peak height (APH) for individual contributors that were present in low template amounts (≤100 pg). Each data point represents the average peak height in RFUs representing all alleles detected for selected individual low-template contributor, regardless of allele sharing or specific mixture ratio, for three-person high share (H), three-person low share (L), four-person, and five-person mixtures. 20 – 25 values of each were selected for the three-, four-, and five-contributor mixtures that demonstrated the range of LRs observed in the study.

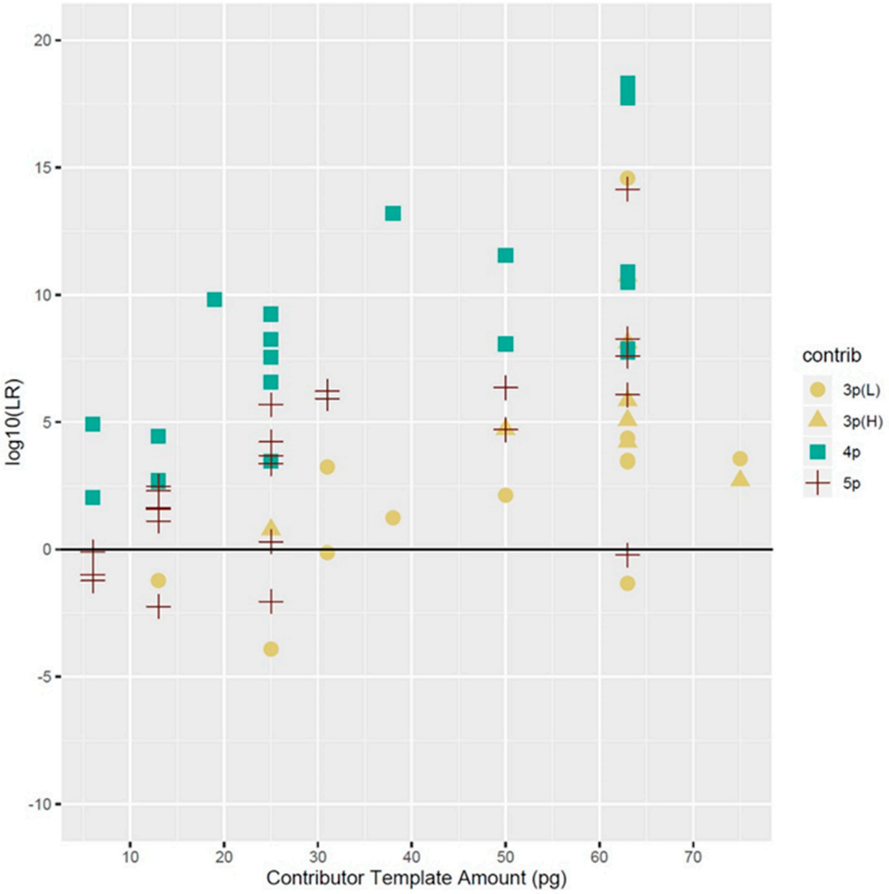

Supplementary Figure S6. The log10 of the average LRs (N=5) versus DNA template quantity (pg) for individual contributors that were present in low template amounts (≤100 pg). The approximate quantities of DNA for individual low-template contributors, regardless of mixture ratio are shown. 20 – 25 values each were selected for the three-, four-, and five-contributor mixtures that demonstrated the range of LRs observed in the study.

Profile: 1669_053018_AlleleReport.txt (Reference) × ▾

Population: All data × ▾

Overall Likelihood Ratio

Calculation	Numerator	Denominator	Likelihood Ratio
**Overall LR (vs Random)**	**2.01e-105**	**1.24e-88**	**1.62e-17**

Marker-Specific Likelihood Ratios

Marker	Genotype	Numerator	Denominator	Likelihood Ratio
D3S1358	15, 15	3.32e-3	1.42e-3	2.34e+0
D1S1656	18.3, 18.3	2.42e-7	9.47e-6	2.56e-2
D2S441	10, 14	1.95e-4	1.07e-3	1.83e-1
D10S1248	13, 14	3.56e-2	2.95e-3	1.21e+1
D13S317	11, 12	1.08e-5	1.19e-4	9.05e-2
Penta_E	10, 11	1.25e-7	2.54e-6	4.92e-2
D16S539	11, 12	3.58e-4	5.34e-5	6.69e+0
D18S51	15, 16	1.78e-4	3.43e-5	5.17e+0
D2S1338	17, 24	3.22e-6	6.13e-6	5.25e-1
CSF1PO	10, 11	4.15e-3	1.08e-3	3.85e+0
Penta_D	9, 12	9.06e-6	1.29e-5	7.05e-1
TH01	9.3, 9.3	1.51e-4	4.62e-4	3.26e-1
vWA	15, 17	1.90e-5	4.65e-5	4.09e-1
D21S11	30, 33.2	1.48e-3	2.56e-5	5.79e+1
D7S820	9, 11	1.71e-3	5.64e-4	3.04e+0
D5S818	11, 13	4.27e-3	1.01e-3	4.23e+0
TPOX	8, 9	2.25e-6	1.33e-5	1.69e-1
D8S1179	11, 14	2.85e-3	9.96e-5	2.87e+1
D12S391	17, 18	1.47e-4	7.74e-6	1.90e+1
D19S433	14, 14	1.68e-2	1.40e-3	1.20e+1
FGA	18, 21	1.96e-5	7.75e-6	2.53e+0
D22S1045	15, 15	7.18e-3	5.97e-4	1.20e+1
**Simple LR (Multiply Markers)**		**4.01e-84**	**4.13e-89**	**9.72e+4**

Supplementary Figure S7. Reported LRs for contributor 1669 in a four-person mixture with a 1:2:2:5 ratio (~100:200:200:500 pg) as displayed in MaSTR™ when analyzed with N-1 true contributors. The LR values for 1669 shown are for one of the five replicate analyses conducted and so differ from the averaged values shown in Table 5. Contributor 1669 occupied the first position in this mixture, [1]:2:2:5 (~100 pg), and no allele drop-out was observed for it.

Profile: 1682_053018_AlleleReport.txt (Alternate) × ▾

Population: All data × ▾

Overall Likelihood Ratio

Calculation	Numerator	Denominator	Likelihood Ratio
**Overall LR (vs Random)**	**1.73e-75**	**1.24e-88**	**1.40e+13**

Marker-Specific Likelihood Ratios

Marker	Genotype	Numerator	Denominator	Likelihood Ratio
D3S1358	15, 17	1.40e-2	1.42e-3	9.87e+0
D1S1656	14, 15	7.71e-6	9.47e-6	8.13e-1
D2S441	11, 11	3.88e-3	1.07e-3	3.64e+0
D10S1248	13, 14	3.56e-2	2.95e-3	1.21e+1
D13S317	12, 12	2.57e-4	1.19e-4	2.16e+0
Penta_E	7, 12	2.37e-5	2.54e-6	9.33e+0
D16S539	10, 14	5.33e-3	5.34e-5	9.97e+1
D18S51	13, 15	3.72e-4	3.43e-5	1.08e+1
D2S1338	17, 18	3.00e-4	6.13e-6	4.89e+1
CSF1PO	10, 12	2.81e-3	1.08e-3	2.61e+0
Penta_D	5, 11	1.35e-4	1.29e-5	1.05e+1
TH01	7, 9.3	9.40e-5	4.62e-4	2.04e-1
vWA	17, 19	1.12e-3	4.65e-5	2.41e+1
D21S11	30, 31.2	6.31e-4	2.56e-5	2.46e+1
D7S820	9, 11	1.71e-3	5.64e-4	3.04e+0
D5S818	10, 12	7.46e-3	1.01e-3	7.41e+0
TPOX	7, 9	2.59e-3	1.33e-5	1.95e+2
D8S1179	12, 12	1.73e-5	9.96e-5	1.74e-1
D12S391	17, 18	1.47e-4	7.74e-6	1.90e+1
D19S433	13, 13	5.54e-3	1.40e-3	3.96e+0
FGA	21, 23	3.20e-4	7.75e-6	4.14e+1
D22S1045	15, 17	1.09e-2	5.97e-4	1.83e+1
**Simple LR (Multiply Markers)**		**2.22e-69**	**4.13e-89**	**5.38e+19**

Supplementary Figure S8. Reported LRs for contributor 1682 in a four-person mixture with a 1:2:2:5 ratio (~100:200:200:500 pg) as displayed in MaSTR™ when analyzed with N-1 true contributors. The

LR values for 1682 shown are for one of the five replicate analyses conducted and so differ from the averaged values shown in Table 5. Contributor 1682 occupied the second position in this mixture, 1:[2]:2:5 (~200 pg), and no allele drop-out was observed for it.

Profile: 1683_053018_AlleleReport.txt (Alternate)

Population: All data

Overall Likelihood Ratio

Calculation	Numerator	Denominator	Likelihood Ratio
**Overall LR (vs Random)**	**1.29e-94**	**1.24e-88**	**1.04e-6**

Marker-Specific Likelihood Ratios

Marker	Genotype	Numerator	Denominator	Likelihood Ratio
D3S1358	16, 17	2.31e-3	1.42e-3	1.63e+0
D1S1656	13, 17.3	9.13e-8	9.47e-6	9.64e-3
D2S441	10, 10	1.58e-2	1.07e-3	1.48e+1
D10S1248	13, 15	2.12e-2	2.95e-3	7.21e+0
D13S317	8, 13	1.17e-3	1.19e-4	9.79e+0
Penta_E	7, 15	1.03e-6	2.54e-6	4.05e-1
D16S539	11, 11	2.86e-4	5.34e-5	5.35e+0
D18S51	16, 17	1.54e-6	3.43e-5	4.47e-2
D2S1338	18, 24	4.31e-4	6.13e-6	7.04e+1
CSF1PO	11, 12	1.71e-3	1.08e-3	1.58e+0
Penta_D	9, 12	9.06e-6	1.29e-5	7.05e-1
TH01	6, 9.3	8.51e-3	4.62e-4	1.84e+1
vWA	18, 19	1.07e-5	4.65e-5	2.31e-1
D21S11	29, 30	1.33e-6	2.56e-5	5.19e-2
D7S820	10, 12	1.24e-3	5.64e-4	2.20e+0
D5S818	11, 13	4.27e-3	1.01e-3	4.23e+0
TPOX	10, 11	1.71e-4	1.33e-5	1.29e+1
D8S1179	13, 13	1.45e-7	9.96e-5	1.45e-3
D12S391	17, 23	6.21e-4	7.74e-6	8.02e+1
D19S433	14, 14	1.68e-2	1.40e-3	1.20e+1
FGA	21, 23	3.20e-4	7.75e-6	4.14e+1
D22S1045	15, 17	1.09e-2	5.97e-4	1.83e+1
**Simple LR (Multiply Markers)**		**1.45e-82**	**4.13e-89**	**3.52e+6**

Supplementary Figure S9. Reported LRs for contributor 1683 in a four-person mixture with a 1:2:2:5 ratio (~100:200:200:500 pg) as displayed in MaSTR™ when analyzed with N-1 true contributors. The LR values for 1683 shown are for one of the five replicate analyses conducted and so differ from the averaged values shown in Table 5. Contributor 1683 occupied the third position in this mixture, 1:2:[2]:5 (~200 pg), and no allele drop-out was observed for it.

Profile: 1690_053018_AlleleReport.txt (Alternate)

Population: All data

Overall Likelihood Ratio

Calculation	Numerator	Denominator	Likelihood Ratio
Overall LR (vs Random)	2.24e-63	1.24e-88	1.81e+25

Marker-Specific Likelihood Ratios

Marker	Genotype	Numerator	Denominator	Likelihood Ratio
D3S1358	15, 16	8.23e-3	1.42e-3	5.80e+0
D1S1656	16, 17.3	3.17e-4	9.47e-6	3.35e+1
D2S441	11, 14	6.63e-3	1.07e-3	6.22e+0
D10S1248	13, 14	3.56e-2	2.95e-3	1.21e+1
D13S317	10, 12	3.32e-3	1.19e-4	2.78e+1
Penta_E	14, 14	1.88e-4	2.54e-6	7.41e+1
D16S539	11, 12	3.58e-4	5.34e-5	6.69e+0
D18S51	15, 17	7.71e-4	3.43e-5	2.25e+1
D2S1338	20, 25	2.70e-4	6.13e-6	4.41e+1
CSF1PO	11, 11	1.69e-2	1.08e-3	1.57e+1
Penta_D	10, 13	3.68e-4	1.29e-5	2.86e+1
TH01	6, 6	5.78e-3	4.62e-4	1.25e+1
vWA	18, 18	1.37e-3	4.65e-5	2.94e+1
D21S11	29, 29	5.91e-4	2.56e-5	2.31e+1
D7S820	10, 11	6.09e-3	5.64e-4	1.08e+1
D5S818	11, 12	6.30e-3	1.01e-3	6.26e+0
TPOX	8, 8	5.98e-5	1.33e-5	4.50e+0
D8S1179	12, 15	2.49e-3	9.96e-5	2.50e+1
D12S391	16, 19	6.26e-4	7.74e-6	8.09e+1
D19S433	14, 14	1.68e-2	1.40e-3	1.20e+1
FGA	18, 23	1.72e-3	7.75e-6	2.22e+2
D22S1045	15, 15	7.18e-3	5.97e-4	1.20e+1
**Simple LR (Multiply Markers)**		**6.21e-61**	**4.13e-89**	**1.50e+28**

Supplementary Figure S10. Reported LRs for contributor 1690 in a four-person mixture with a 1:2:2:5 ratio (~100:200:200:500 pg) as displayed in MaSTR™ when analyzed with N-1 true contributors. The LR values for 1690 shown are for one of the five replicate analyses conducted and so differ from the averaged values shown in Table 5. Contributor 1690 occupied the fourth position in this mixture, 1:2:2:[5] (~500 pg), and no allele drop-out was observed for it.
